# Blocking Metabotropic Glutamate Receptor Subtype 7 via the Venus Flytrap Domain Promotes a Chronic Stress-Resilient Phenotype in Mice

**DOI:** 10.3390/cells11111817

**Published:** 2022-06-02

**Authors:** Karolyne A. R. Estrela, Lisa Senninger, Josephine Arndt, Melanie Kabas, Ferdinand Schmid, Larissa Dillmann, Sophia Auer, Thomas Stepfer, Peter J. Flor, Nicole Uschold-Schmidt

**Affiliations:** Laboratory of Molecular and Cellular Neurobiology, Faculty of Biology and Preclinical Medicine, University of Regensburg, 93053 Regensburg, Germany; karolyne.estrela@biologie.uni-regensburg.de (K.A.R.E.); senninger.lisa@web.de (L.S.); josephine.arndt@osc-arndt.com (J.A.); melanie.kabas@stud.uni-regensburg.de (M.K.); ferdi.schmid@gmx.de (F.S.); larissa_dillmann@live.com (L.D.); sophia-auer@gmx.de (S.A.); tomstefper@gmx.de (T.S.)

**Keywords:** mGlu7, XAP044, chronic psychosocial stress, chronic subordinate colony housing

## Abstract

Chronic psychosocial stress participates prominently in the etiology of various psychiatric conditions and comorbid somatic pathologies; however, suitable pharmacotherapy of these disorders is still of high medical need. During the last few decades, research on mGlu receptors advanced remarkably and much attention was given to the mGlu7 subtype. Here, genetic mGlu7 ablation, short-term pharmacological mGlu7 blockade, as well as siRNA-mediated knockdown of mGlu7 were shown to result in an acute anti-stress, antidepressant- and anxiolytic-like phenotype in mice. Moreover, we recently revealed a prominent stress-protective effect of genetic mGlu7 ablation also with respect to chronic psychosocial stress. In addition, we are able to demonstrate in the present study that the chronic pharmacological blockade of mGlu7 interferes with various chronic stress-induced alterations. For this, we used the chronic subordinate colony housing (CSC), a mouse model of chronic male subordination, in combination with chronic treatment with the mGlu7-selective orthosteric-like antagonist XAP044 (7-hydroxy-3-(4-iodophenoxy)-4H-chromen-4-one). Interestingly, XAP044 dose-dependently ameliorates hypothalamic–pituitary–adrenal axis dysfunctions, thymus atrophy, as well as the CSC-induced increase in innate anxiety. Taken together, our findings provide further evidence for the role of mGlu7 in chronic psychosocial stress-induced alterations and suggests the pharmacological blockade of mGlu7 as a promising therapeutic approach for the treatment of chronic stress-related pathologies in men.

## 1. Introduction

Chronic stress-related psychiatric conditions and comorbid somatic pathologies are an enormous public health concern in modern society. The etiology of these disorders is complex, with stressors holding a chronic and psychosocial component that represent the most acknowledged risk factors [1,2,3]. To date, there is still a dearth of knowledge about the underlying physiological, neural, and immunological mechanisms linking chronic stress with such disorders and treatment options are insufficient. The L-glutamatergic system represents the primary excitatory neurotransmitter system of the mammalian brain and consists of a diverse family of receptors broadly divided into ionotropic glutamate receptors (iGlu) and metabotropic glutamate receptors (mGlu). During the last few decades, research on mGlu receptors advanced remarkably and much attention was given to the mGlu7 subtype in the context of acute stress-related behavior and physiology [4]. The mGlu7 subtype belongs to the group III of the metabotropic glutamate receptor family of G protein-coupled receptors, consisting of an N-terminal extracellular domain with cysteine-rich regions and a Venus flytrap domain (VFTD). The large N-terminal extracellular domain is connected to a seven-transmembrane region (∼260 amino acids), followed by an intracellular C-terminal domain consisting of 30–300 amino acids [5]. It shows the highest degree of evolutionary conservation within the family and is the most widely distributed mGlu family member mainly localized on presynaptic terminals of glutamatergic and GABAergic neurons, where it is thought to regulate neurotransmitter release [6,7]. Besides key brain regions involved in stress perception and the stress response, such as the neocortex, hippocampus, amygdala, locus coeruleus, thalamus and hypothalamus [7,8,9], the expression of mGlu7 was also reported in the olfactory bulb and in peripheral organs, such as the adrenal glands, the colon and stomach [10,11,12]. Interestingly, genetic mGlu7 ablation [13,14], pharmacological mGlu7 blockade [15,16,17], as well as siRNA-mediated knockdown of mGlu7 [18,19] were shown to result in an acute anti-stress, antidepressant- and anxiolytic-like phenotype in mice. These data provide clear evidence for a prominent role of mGlu7 in acute stress-related behavior and physiology. Because less attention was given to the role of mGlu7 in chronic stress-related affective and somatic disorders, we recently addressed the effects of chronic subordinate colony housing (CSC), a mouse model of chronic psychosocial stress in male mice, on the brain glutamatergic system. Notably, the CSC paradigm represents a valuable animal model for male mice as it mimics the type of health compromising stressors of human daily life through a combination of chronic, psychological, and social aspects of stress. CSC exposure reliably leads to both somatic and affective consequences, including systemic inflammation, stress axes dysfunctions and increased anxiety-related behavior; thus, it represents a powerful model to study the mechanisms underlying various chronic stress-induced pathologies [3,20,21]. Beside these well-described effects, 19 days of CSC resulted in a significant downregulation of central mGlu7 mRNA expression restricted to the prefrontal cortex. This outcome indicates region-specific modulation of the mGlu7 gene in response to chronic psychosocial stress [22]. Using the CSC model in combination with mGlu7 knockout mice, we were additionally able to demonstrate a chronic stress-protective phenotype in mice with genetic mGlu7 ablation exposed to chronic psychosocial stress. Beside protection against the CSC-induced anxiety-prone phenotype, mGlu7-deficient mice were also less vulnerable to CSC, with respect to physiological and immunological alterations. For example and in contrast to their wildtype littermates, mGlu7-deficient mice showed no CSC-induced stress axis dysfunctions and systemic inflammation. Together, these findings point to a distinct role of mGlu7 in modulating a wide range of affective and somatic alterations that occur upon chronic psychosocial stressor exposure induced by CSC [22]. Apart from genetic mGlu7 ablation, pharmacological mGlu7 blockade represents the most suitable independent approach to obtain additional evidence for the involvement of the mGlu7 receptor subtype in chronic psychosocial stress-induced somatic and affective consequences using the CSC paradigm and to shed more light on the underlying mechanisms. To this end, the first orthosteric-like mGlu7 antagonist XAP044, discovered in collaboration with our own group, represents an adequate and promising pharmacological tool. In detail, XAP044 is a low-micromolar potent antagonist at mGlu7 (IC_50_ 1–4 µM, depending on the functional assay used), with 10-fold or higher selectivity against related receptors (mGlu- and GABA_B_-receptors) and also against rather unrelated targets, such as vasopressin 1a and oxytocin receptors; XAP044’s selectivity for mGlu7 is independent of the assay used. Interestingly, XAP044 binds within the VFTD close to the binding site of the orthosteric agonist L-glutamate of mGlu7, thereby blocking the orthosteric ligand-induced closing of the two lobes of the VFTD and, hence, the propagation of the signal via the extracellular cysteine-rich domain and the transmembrane and cytoplasmic domains. XAP044 was already shown to selectively block long-term potentiation in the lateral amygdala in an mGlu7-dependent manner and to have acute anti-stress and antidepressant efficacy in rodent behavioral paradigms [15]. Based on our previous findings, we hypothesized that the pharmacological blockade of mGlu7 using XAP044 may control the vulnerability to chronic psychosocial stress. To test this, we used male mice chronically infused (*i.c.v.*) with different doses of XAP044 in combination with the CSC paradigm (for 19 days).

## 2. Materials and Methods

### 2.1. Animals

Male C57BL/6 mice (Charles River, Sulzfeld, Germany), all weighing 19–22 g, were used as experimental mice and individually housed in standard polycarbonate mouse cages (16 × 22 × 14 cm) for at least one week before starting the CSC procedure. Male CD1 mice weighing 30–35 g from our own breeding were used as dominants. All mice were kept under standard laboratory conditions (12 h light/dark cycle, lights on at 0600 h, 22 °C, 60% humidity) with free access to tap water and standard mouse diet (ssniff Spezialdiäten GmbH, Soest, Germany). All experimental protocols were approved by the Committee on Animal Health and Care of the local government and conformed to international guidelines on the ethical use of animals. All efforts were made to minimize animals suffering and to reduce the number of animals used.

### 2.2. Experimental Design

**Figure 1:**
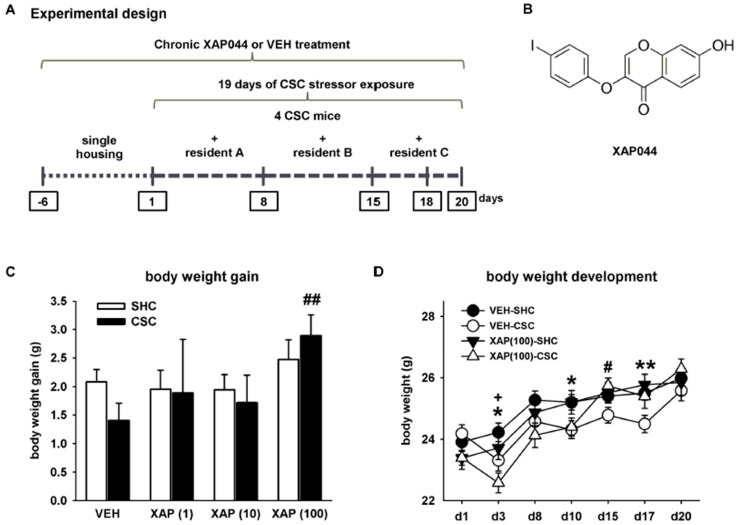
Experimental design of the chronic subordinate colony housing (CSC, 19 days) paradigm and effects of CSC exposure and *i.c.v.* drug-treatment on body weight. (**A**) Schematic illustration of the CSC paradigm. (**B**) Chemical structure of the used mGlu7-selective orthosteric-like antagonist XAP044. During 19 days of CSC, (**C**) body weight gain (day 20–day 1) (factor housing: F_1,147_ = 0.197, *p* = 0.658; factor treatment: F_3,147_ = 2.102, *p* = 0.102; housing*treatment interaction: F_3,147_ = 0.718, *p* = 0.543) and (**D**) body weight development (factor time: F_6,528_ = 104.692, *p* ≤ 0.001; factor treatment: F_3,88_ = 1.176, *p* = 0.323; time*treatment interaction: F_18,528_ = 5.979, *p* ≤ 0.001) were assessed in SHC and CSC mice treated either with VEH (5% DMSO) or XAP044 at different doses. White bar, SHC; black bar, CSC. *n* = 7–35 per treatment and housing group. Data represent the mean ± SEM. * *p* ≤ 0.05, ** *p* ≤ 0.01 VEH-CSC vs. VEH-SHC in (**D**); + *p* ≤ 0.05 XAP(100)-CSC vs. XAP(100)-SHC in (**D**); # *p* ≤ 0.05, ## *p* ≤ 0.01 XAP(100)-CSC vs. VEH-CSC in (**C**,**D**); two-way ANOVA followed by Bonferroni *post hoc* analysis or independent Student’s *t*-test (**C**) or repeated measures ANOVA, followed by Bonferroni *post hoc* analysis or independent Student’s *t*-test (**D**).

To assess the effects of chronic pharmacological mGlu7 blockade on chronic stress-induced alterations, experimental mice (*n* = 6–36 per housing or treatment group, depending on the number of animals used per experiment and the number of experiments performed/pooled) received a micro-osmotic pump (Alzet^®^, Model 1004, 0.11 µL/h, Cupertino, CA, USA) implanted *s.c.* in the abdominal region and connected to an *i.c.v.* infusion cannula to enable chronic *i.c.v.* administration of XAP044 at different doses (100 µM, 10 µM and 1 µM) or vehicle (5% DMSO in Ringer’s solution). One week after surgery, mice were either chronically stressed by exposure to the CSC paradigm or single-housed for control (SHC) in a treatment- and weight-matched setup. The CSC paradigm lasted for 19 consecutive days and was conducted as described elsewhere [20,22,23] and in detail below (see Figure 1A).

### 2.3. Chronic Subordinate Colony Housing (CSC) Paradigm

The CSC paradigm was conducted as described previously [3,4,20]. Briefly, experimental mice were assigned to the single-housed control (SHC) or chronic subordinate colony housing (CSC) group in a treatment- and weight-matched manner. Four CSC mice of the same treatment were housed together with a dominant male for 19 consecutive days in order to induce chronic psychosocial stress. To avoid habituation during chronic stressor exposure, each dominant male was replaced by a novel dominant male on days 8 and 15. As appropriate controls, SHC mice were used [24], being in line with previous studies demonstrating single housing to be less stressful in male mice as compared to group housing [25,26,27]. SHC mice remained undisturbed in their home cage except for the change of bedding once a week. On day 15 of CSC, anxiety-related behavior was assessed in the light-dark box (LDB) test. On day 18 of CSC, physiological anxiety was assessed using the stress-induced hyperthermia test (SIH). After testing, stressed mice were placed back into their respective CSC colony and SHC mice remained single-housed. On day 20, all mice were rapidly decapitated between 0800 h and 1100 h.

### 2.4. Drug Treatment/Surgical Procedure

Chronic application of the orthosteric-like mGlu7 antagonist XAP044 (Tocris Bioscience, Bristol, UK; Figure 1B) via *s.c.*-implanted Alzet^®^ micro-osmotic pumps (pumping rate: 0.11 µL/h, Alzet^®^, Model 1004, Cupertino, CA, USA) connected to an *i.c.v.* infusion cannula (Brain Infusion Kit 3, Alzet^®^, Cupertino, CA, USA) was initiated one week before starting the CSC paradigm (day 6) in order to establish a stable baseline receptor occupancy and was continued until the end of chronic stressor exposure (day 20). XAP044 was formulated as a solution in vehicle (VEH, 5% DMSO in Ringer’s solution, Merck; Darmstadt, Germany) to ensure a continuous substance release of 1 µM, 10 µM or 100 µM of XAP044. As reported by Peterlik et al. 2017 [23], the micro-osmotic pump was implanted *s.c.* in the abdominal region through a 1 cm long incision at the lower neck of the mouse under isoflurane anesthesia (Baxter, GmbH, Unterschleißheim, Germany) but additionally connected via a catheter to an *i.c.v.* infusion cannula. Animals were treated with painkiller (Buprenovet, *s.c.*; 0.05 mg/kg; Bayer Health Care AG, Leverkusen, Germany) and 100 µL of antibiotics (*s.c.*, Baytril^®^, 2.5% Bayer Health Care AG, Leverkusen, Germany). Wound treatment was conducted using betaisodona (Mundipharma GmbH, Limburg, Germany).

### 2.5. Light-Dark Box (LDB) Test

To assess the treatment-specific effects of CSC on anxiety-related behavior, SHC and CSC mice were tested in the LDB (day 15) [23] between 0800 h and 1100 h. CSC mice were directly taken from the colony cages without single housing prior to LDB testing. Afterwards, CSC mice were put back in their respective CSC colony and SHC mice were kept single housed. The LDB consisted of a bright (27 × 27 × 27 cm; 140 lux) and a dark (18 × 27 × 27 cm; 20 lux) compartment, separated by a partition wall that had a small opening (6 × 6 cm high) at floor level. For habituation, mice were individually placed in the dark compartment, with the opening of the partition wall closed for 30 s. Afterwards, the partition wall was opened and mice were allowed to freely explore the arena for 5 min. The time spent in the bright compartment (as a measure of anxiety) and the number of line crossings (as a measure of locomotor activity) were analyzed by an observer blind to the animal’s housing and treatment condition. The LDB was cleaned thoroughly before each test.

### 2.6. Stress-Induced Hyperthermia (SIH) Test

On day 18, the SIH test was performed as described elsewhere [15,19,23] between 0800 h and 1100 h. Rectal temperature was recorded twice at T1 and T2 (15 min later) using a digital thermometer (2.5 mm diameter, Amarell GmbH and Co, KG, Kreuzwertheim, Germany). In order to avoid any rectal injuries, the thermometer was covered with milking grease before inserting it 1.5 cm into the rectum. Recording of T1 indicated baseline temperature and served as stressor at the same time. Recording of T2 allowed determination of the SIH response defined as the difference between T2 and T1.

### 2.7. Determination of Body Weight and Organ Weight

On day 20, mice were weighed immediately before decapitation to assess the effects of CSC and treatment on body weight. Afterwards, the pituitary, left and right adrenal glands, thymus, and spleen of each animal were removed, pruned from fat and weighed separately. In addition, the sum of left and right absolute adrenal weights was calculated for each animal. Until all mice were killed and adrenals removed, the latter were stored in ice-cold DMEM (DMEM/F-12, Life Technologies, Darmstadt, Germany) containing 0.1% bovine serum albumin (BSA). Values represent absolute measurements (in mg) of the respective organs.

### 2.8. Trunk Blood Sampling

To determine the effects of CSC and treatment on basal morning plasma CORT concentrations, trunk blood was also collected from all experimental animals after rapidly killing by decapitation under CO_2_ anesthesia within 3 min after entering the animal room between 800 h and 1100 h. Trunk blood was collected in EDTA-coated tubes (Sarstedt, Nuembrecht, Germany) on ice and centrifuged at 4 °C (5000 rpm, 5 min). Plasma samples were stored at −20 °C until assayed.

### 2.9. ACTH Stimulation of Adrenal Explants In Vitro

Stimulation of adrenal explants with ACTH (100 nM) in vitro was performed as previously described [21,28]. Briefly, left and right adrenals were stored in ice-cold DMEM/F-12 (Life Technologies, Darmstadt, Germany), containing 0.1% BSA until all mice were killed and adrenals removed. Afterwards, each left and right adrenal gland was cut into two halves, each containing cortical and medullary tissue. The halves were then weighed and pre-incubated in 200 µL of DMEM/F-12 for 4 h (37 °C, 5% CO_2_) before any further treatment. Culture medium was then replaced, and each half of one adrenal was incubated with medium containing either 0.9% saline (basal) or 0.9% saline plus ACTH (100 nM) for 6 h (37 °C, 5% CO_2_). After incubation, supernatants were carefully removed and stored at −20 °C until being analyzed using a commercially available ELISA for CORT (IBL International, Hamburg, Germany). CORT concentrations were calculated in relation to the weight of the respective adrenal explants (i.e., relative CORT secretion). To illustrate the in vitro adrenal CORT secretion in relation to the whole organism, the relative CORT secretion from the left and right adrenal gland of each mouse was summed up.

### 2.10. ELISA for CORT

Plasma and supernatant samples were analyzed using a commercially available ELISA for CORT (analytical sensitivity < 1.631 nmol/L and intra-assay and inter-assay coefficients of variation (CV) ≤ 6.35%; IBL International, Hamburg, Germany).

### 2.11. Cannula Placement and Remaining Volume in the Micro-Osmotic Pumps

On day 20, blue ink was inserted into the brain after decapitation through the catheter and cannula to prove the correct placement of the cannula in the lateral ventricle. Pumps were removed and the remaining quantity of solved drug or vehicle was assessed to check for proper pump functioning according to the manufacturer (ALZET^®^, Model 1004, Cupertino, CA, USA). Animals without correct placement of the cannula or improper pump functioning were removed from the data analysis.

### 2.12. Statistical Analysis

All data represent the mean + or ± S.E.M. and were analyzed using the software IBM SPSS 28.0 (IBM Corporation, Armonk, NY, USA). Parameters depending on one factor (housing or treatment) were analyzed using independent Student’s *t*-test or one-way analysis of variance (ANOVA), followed by Bonferroni *post hoc* testing. For analyzing parameters depending on two factors (i.e., housing and treatment, housing and stimulation) two-way ANOVA was employed. Significant main and interaction effects were followed by Bonferroni *post hoc* analysis when appropriate or an independent Student’s *t*-test. Data depending on each other were analyzed using repeated measures ANOVA, followed by Bonferroni *post hoc* testing or an independent Student’s *t*-test. Statistical significance was accepted at *p* < 0.05.

## 3. Results

### 3.1. Chronic Pharmacological mGlu7 Blockade Interferes with Multiple CSC-Induced Physiological Alterations and Weight Changes in Lymphatic Organs in a Dose-Dependent Manner

Due to the promising chronic stress-protective phenotype of mGlu7 knockout mice, we further addressed the functional involvement of mGlu7 in chronic stress physiology using chronic pharmacological inhibition of mGlu7 in combination with CSC. Importantly, there were no side effects detectable in the in vivo experiments performed so far, neither with XAP044 given *i.p.* up to the highest dose used of 60 mg/kg [15], nor with XAP044 chronically applied *i.c.v.* up to the highest dose used of 100 µM in the present experiments. Moreover, testing the chronic administration of the vehicle (5% DMSO in Ringer’s solution) and XAP044 (100 µM) *i.c.v*. via micro-osmotic pumps implanted *s.c.* in naïve mice for 26 days excluded any undesirable effects on parameters typically assessed after CSC exposure when compared to the administration of Ringer’s solution (Supplementary Figure S1). This is in line with Peters et al. (2014) and Peterlik et al. (2017) who revealed no confounding influence of either the surgical procedure or the chronic *i.c.v.* administration of vehicle (Ringer’s solution and polyethylene glycol 400, respectively) via micro-osmotic pumps.

In mice implanted with micro-osmotic pumps (*s.c.*) connected to an *i.c.v.* infusion cannula, we could not detect any effects of CSC exposure on body weight gain in all groups assessed. However, CSC mice treated with 100 µM of XAP044 gained more weight during 19 days of CSC, compared to all other CSC groups. This effect was even significant when compared to the VEH-CSC group (independent Student’s *t*-test: *t*_40_ = −2.707, *p* = 0.010; Figure 1C). A closer look at the body weight development of mice treated with VEH or 100 µM of XAP044 (exemplary for all XAP044 doses) during CSC revealed a positive development of body weight over a period of 19 days in all mice, irrespective of treatment and housing. Moreover, independent Student’s *t*-tests revealed a decreased body weight in CSC compared to SHC mice of the vehicle group on day 3 (*t*_63_ = 1.966, *p* = 0.054), day 10 (*t*_63_ = 2.295, *p* = 0.025) and day 17 (*t*_63_ = 2.730, *p* = 0.008). In mice treated with 100 µM of XAP044, this CSC effect was only present on day 3 (*t*_25_ = 2.146, *p* = 0.042) but absent on all other days (Figure 1D). Moreover, body weight was significantly higher in CSC mice treated with 100 µM of XAP044 compared to CSC mice of the VEH group on day 15 (*t*_40_ = 2.143, *p* = 0.038; Figure 1D). Together, these results suggest a stress-protective effect of chronic treatment with 100 µM of XAP044 during chronic psychosocial stressor exposure, with respect to body weight development.

**Figure 2:**
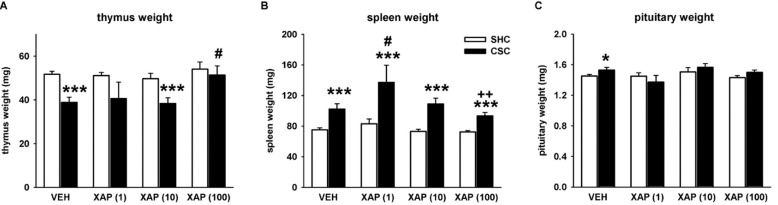
Effects of XAP044 treatment on CSC-induced physiological alterations and weight changes of lymphatic organs. On day 20, (**A**) absolute thymus weight (factor housing: F_1,146_ = 16.481, *p* ≤ 0.001; factor treatment: F_3,146_ = 3.252, *p* = 0.024; housing*treatment interaction: F_3,146_ = 1.135, *p* = 0.337), (**B**) absolute spleen weight (factor housing: F_1,144_ = 43.469, *p* ≤ 0.001; factor treatment: F_3,144_ = 3.259, *p* = 0.023; housing*treatment interaction: F_3,144_ = 1.317, *p* = 0.254) and (**C**) absolute pituitary weight (factor housing: F_1,139_ = 0.762, *p* = 0.384; factor treatment: F_3,139_ = 1.822, *p* = 0.146; housing*treatment interaction: F_3,139_ = 0.699, *p* = 0.554) were assessed in mice treated either with VEH (5% DMSO) or XAP044 at different doses. White bar, SHC; black bar, CSC. *n* = 7–35 per treatment and housing group. Data represent the mean + SEM. * *p* ≤ 0.05, *** *p* ≤ 0.001 vs. respective SHC group; # *p* ≤ 0.05 vs. respective VEH group; ^++^ *p* ≤ 0.05 vs. XAP(1); two-way ANOVA followed by Bonferroni *post hoc* analysis or independent Student’s *t*-test.

CSC housing has also been shown to induce changes in the weight of lymphatic organs. For example, 19 days of CSC exposure typically induce thymus atrophy and splenomegaly [23,29]. Those CSC effects were also present in the VEH group of the present study. In detail, absolute thymus weight was decreased in CSC compared to SHC mice treated with VEH (*p* ≤ 0.001). This CSC-induced thymus atrophy was also present in mice treated with 10 µM (*p* = 0.001), but absent in mice treated with 1 µM and 100 µM of XAP044 (Figure 2A). Moreover, the thymus weight of CSC mice treated with 100 µM was also higher compared to CSC mice treated with VEH (*p* = 0.018), as well as with 10 µM of XAP044 (*p* = 0.016). It should be noted that the absence of an CSC effect in mice treated with 1 µM of XAP044 might be due to the high standard error resulting from the lower number of animals used for the 1 µM-experiments. Together, it seems that at least 100 µM of XAP044 has a stress-protective effect with respect to the typical CSC-induced thymus atrophy.

CSC-induced splenomegaly, i.e., an increase in absolute spleen weight, was detected in all groups of the present study irrespective of treatment (for all groups *p* ≤ 0.001; Figure 2B), with the strongest CSC-induced increase present in mice treated with 1 µM of XAP044. Here, the increase in spleen weight in CSC mice was even significantly higher compared to CSC mice treated with VEH (*p* = 0.019) and 100 µM of XAP044 (*p* = 0.008; Figure 2B). Thus, it seems that XAP044 treatment had no stress-protective effect with respect to the CSC-induced splenomegaly.

Exposure to 19 days of CSC has also been shown to result in profound HPA axis-related changes [20,21,30]. The assessment of absolute pituitary weight revealed a CSC-induced increase in absolute pituitary weight in mice treated with VEH (independent Student’s *t*-test: *t*_58_ = 2.060; *p* = 0.044). This effect was not present in mice treated with XAP044 at all doses tested (Figure 2C).

**Figure 3:**
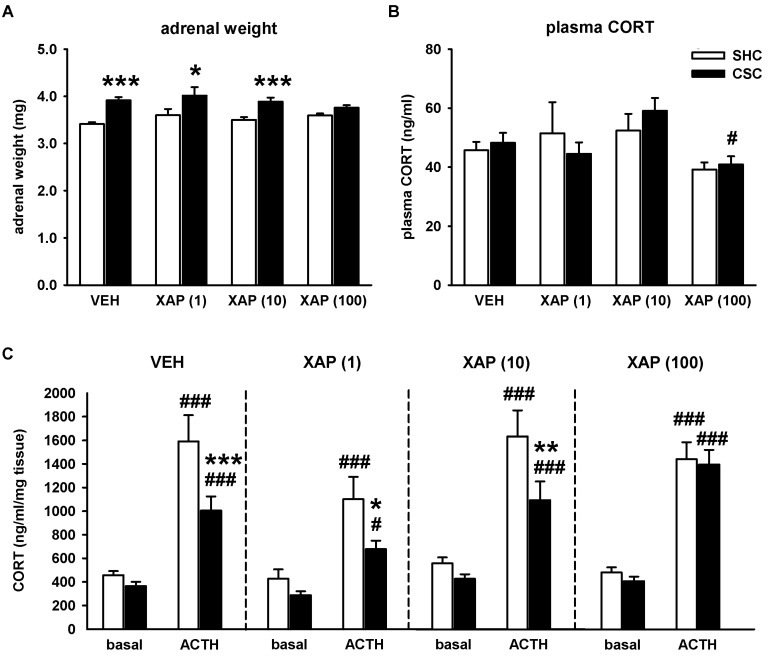
Effects of XAP044 treatment on CSC-induced HPA axis-related changes. On day 20, (**A**) absolute adrenal weight (factor housing: F_1,146_ = 37.888, *p* ≤ 0.001; factor treatment: F_3,146_ = 0.860, *p* = 0.464, housing*treatment interaction: F_3,146_ = 1.783, *p* = 0.153), (**B**) basal morning plasma CORT levels (housing: F_1,137_ = 0.074, *p* = 0.786; factor treatment: F_3,137_ = 4.083, *p* = 0.008; housing*treatment interaction: F_3,137_ = 0.462, *p* = 0.709) and (**C**) adrenal in vitro ACTH responsiveness (VEH: housing: F_1,131_ = 8.337, *p* = 0.005; stimulation: F_1,131_ = 48.465, *p* ≤ 0.001; housing*stimulation interaction: F_1,131_ = 4.583, *p* = 0.034; XAP(1): housing: F_1,26_ = 5.883, *p* = 0.023; stimulation: F_1,26_ = 21.025, *p* ≤ 0.001; housing*stimulation interaction: F_1,26_ = 1.482, *p* = 0.234; XAP(10): housing: F_1,92_ = 6.054, *p* = 0.016; stimulation: F_1,92_ = 39.208, *p* ≤ 0.001; housing*stimulation interaction: F_1,92_ = 2.405, *p* = 0. 124; XAP(100): housing: F_1,48_ = 0.343, *p* = 0.561; stimulation: F_1,48_ = 93.461, *p* ≤ 0.001; housing*stimulation interaction: F_1,48_ = 0.020, *p* = 0.887) were assessed in mice treated either with VEH (5% DMSO) or XAP044 at different doses. White bar, SHC; black bar, CSC. *n* = 6–35 per treatment and housing group. Data represent the mean + SEM. * *p* ≤ 0.05, ** *p* ≤ 0.01, *** *p* ≤ 0.001 vs. respective SHC group; # *p* ≤ 0.05 vs. XAP(10)-CSC in (**B**), # *p* ≤ 0.05, ### *p* ≤ 0.001 vs. respective basal stimulation in (**C**); two-way ANOVA followed by Bonferroni *post hoc* analysis or independent Student’s *t*-test.

CSC exposure also induced an increase in absolute adrenal weight in mice treated with VEH (*p* ≤ 0.001), 1 µM (*p* = 0.013) and 10 µM of XAP044 (*p* ≤ 0.001). This effect was abolished by treatment with 100 µM of XAP044 (Figure 3A).

Basal morning plasma CORT levels were not influenced by CSC exposure in all the groups assessed (Figure 3B). However, mice treated with 100 µM of XAP044 had the lowest level of plasma CORT, with even significantly lower levels in CSC mice treated with 100 µM of XAP044 compared to CSC mice treated with 10 µM of XAP044 (*p* = 0.043; Figure 3B).

We further analyzed the potential treatment-dependent effects of CSC exposure on adrenal ACTH responsiveness *in vitro*. In all groups, adrenal explants from both SHC (*p* ≤ 0.001 for each) and CSC (VEH: *p* ≤ 0.001; 1 µM XAP044: *p* = 0.029; 10 µM XAP044: *p* = 0.001; 100 µM XAP044: *p* ≤ 0.001) mice showed an increased CORT secretion in response to ACTH compared to basal (saline) stimulation. However, in mice treated with VEH, 1 µM or 10 µM of XAP044, ACTH-induced adrenal CORT secretion was lower in CSC compared to SHC mice (VEH: *p* ≤ 0.001; 1 µM XAP044: *p* = 0.016; 10 µM XAP044: *p* = 0.006). This CSC-induced attenuation of adrenal in vitro ACTH responsiveness was abolished in mice treated with 100 µM of XAP044 (Figure 3C). Together, the results indicate a chronic stress-protective effect of XAP044, at least at the dose of 100 µM, with respect to CSC-induced HPA axis-related dysfunctions.

### 3.2. Chronic Pharmacological mGlu7 Blockade Dose-Dependently Reverses CSC-Induced Innate but Not Physiological Anxiety

**Figure 4:**
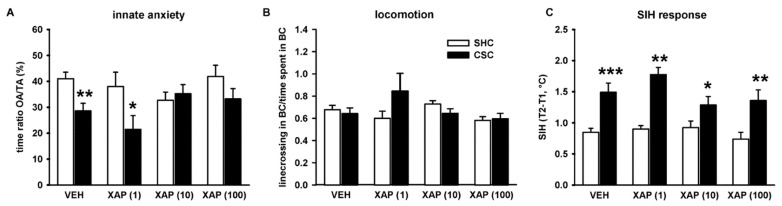
Effects of XAP044 treatment on CSC-induced behavioral changes. In mice treated either with VEH (5% DMSO) or XAP044 at different doses, the percentage of time spent in the bright compartment indicative for the level of innate anxiety (**A**; factor housing: F_1,147_ = 9.187, *p* = 0.003; factor treatment: F_3,147_ = 0.852, *p* = 0. 468; housing*treatment interaction: F_3,147_ = 2.624, *p* = 0.053) and the number of line crossings in the BC indicative for locomotor activity (**B**; factor housing: F_1,147_ = 0.837, *p* = 0.362; factor treatment: F_3,147_ = 1.600, *p* = 0.192; housing*treatment interaction: F_3,147_ = 2.248, *p* = 0.085) were assessed on day 15 of CSC in the LDB. On day 18, the SIH response indicative for the level of physiological anxiety was assessed (**C**; factor housing: F_1,147_ = 36.204, *p* ≤ 0.001; factor treatment: F_3,147_ = 0.987, *p* = 0.401; housing*treatment interaction: F_3,147_ = 1.001, *p* = 0.394). White bar, SHC; black bar, CSC. *n* = 8–36 per treatment and housing group. Data represent the mean + SEM. * *p* ≤ 0.05, ** *p* ≤ 0.01, *** *p* ≤ 0.001 vs. respective SHC group (**A**); two-way ANOVA followed by Bonferroni *post hoc* analysis.

CSC exposure reliably increases anxiety-related behavior in mice [3,23,31]. Therefore, we assessed the possible beneficial effects of chronic XAP044 treatment on CSC-induced innate, as well as physiological anxiety in the LDB (day 15) and SIH test (day 18), respectively. The LDB test revealed decreased time spent in the bright compartment (BC) of the LDB in mice treated with VEH (*p* = 0.002), as well as with 1 µM of XAP044 (*p* = 0.036), which indicates an increase in anxiety-related behavior. This CSC effect was absent in mice treated with 10 µM and 100 µM of XAP044 (Figure 4A). There were no differences between SHC and CSC mice of all groups in the number of line crossings in the BC, suggesting no adverse effects of CSC or XAP044 treatment on locomotor activity (Figure 4B).

With respect to the SIH test, ANOVA revealed a CSC-induced increase in the hyperthermic response in mice irrespective of treatment (VEH: *p* ≤ 0.001; 1 µM XAP044: *p* = 0.002; 10 µM XAP044: *p* = 0.027; 100 µM XAP044: *p* = 0.005; Figure 4C), which indicates an increase in physiological anxiety. Together, these results suggest a dose-dependent beneficial effect of XAP044 treatment on innate but not physiological anxiety in mice.

## 4. Discussion

In the present study, we provide further evidence for the involvement of the mGlu7 receptor subtype in mediating behavioral and physiological alterations, as well as changes in lymphatic organs in response to chronic psychosocial stress in rodents. In addition to the previously published chronic stress-protective phenotype of mice with genetic mGlu7 ablation, we are able to demonstrate a chronic stress-protective effect of chronic pharmacological mGlu7 blockade in mice exposed to CSC (a chronic subordination paradigm for male mice). Chronic *i.c.v.* administration of the orthosteric-like mGlu7-selective antagonist XAP044 during 19 days of chronic stressor exposure dose-dependently attenuated or even prevented prominent CSC-induced behavioral, physiological and lymphatic organs changes. Therefore, our findings suggest that mGlu7 pharmacological blockers have to be considered as a relevant option for the treatment of chronic stress-related emotional and somatic dysfunctions in man.

Given the robust chronic stress-protective phenotype of mGlu7 KO mice [22], we further analyzed the effects of pharmacological mGlu7 blockade in mice exposed to CSC. Relevant CSC-affected parameters were analyzed in detail after chronic (during CSC) *i.c.v.* administration of the orthosteric-like mGlu7-selective antagonist XAP044. Different doses of XAP044 (1 µM, 10 µM and 100 µM) were used to evaluate a possible dose-dependency. The doses used were chosen according to previous published data, suggesting sufficient brain exposure and receptor occupancy as well as activity in animal models of anxiety and maternal behavior [15,32]. Importantly, chronic *i.c.v.* administration of the vehicle (5% DMSO in Ringer’s solution), as well as the highest dose of XAP044 (100 µM) compared to Ringer’s solution alone, were tested beforehand in naïve mice to exclude any confounding influence of either the surgical procedure or of the chronic administration itself (please see Supplementary Figure S1).

### 4.1. Chronic Pharmacological mGlu7 Blockade Reverses Multiple CSC-Induced Physiological Alterations and Weight Changes of Lymphatic Organs

Well in line with our previous studies using untreated mice or mice chronically treated *s.c.* or *i.p.* with VEH [21,22,23], *i.c.v.*-treated CSC vs. SHC mice of the VEH group of the present study showed typical reliable CSC-induced physiological and lymphatic organs alterations, following 19 days of CSC. They showed a decrease in body weight, especially two days after the onset of CSC on day 3, as well as two days after changing the residents on days 10 and 17 [23]. Moreover, they showed alterations in HPA axis functionality, including hypertrophy of the pituitary and of the adrenal glands, together with a reduced in vitro ACTH responsiveness of the latter. In addition, the well-described CSC-induced thymus atrophy and splenomegaly were also present in CSC compared to SHC mice of the VEH group [3,21,23,28,29].

Interestingly, most of these CSC-induced physiological and lymphatic organs changes found in VEH-treated mice were attenuated or even abolished in mice chronically treated with XAP044 *i.c.v.*, with the effects being strongly dose-dependent. For example, although the body weight gain of SHC and CSC mice treated with 1 µM and 10 µM of XAP044 was comparable to the weight gain of the respective VEH group, the highest level of body weight gain was found in CSC mice treated with 100 µM of XAP044. Moreover, in mice treated with 100 µM of XAP044, a CSC-induced decrease in body weight was only detectable on day 3 of CSC. Treatment with 100 µM of XAP044 was able to block the CSC-induced decrease in body weight found in VEH-treated mice on day 10 and 17. Furthermore, the body weight of CSC mice treated with 100 µM of XAP044 was even significantly higher compared to CSC mice of the VEH group on day 15 of CSC, supporting a stress-protective effect of XAP044 treatment with respect to body weight development.

A stress-protective effect of XAP044 treatment was also found regarding the weight of lymphatic organs. XAP044 dose-dependently attenuated the CSC-induced thymus atrophy, with the decrease in thymus weight being completely abolished by treatment with 100 µM of XAP044. Interestingly, it seems that XAP044 treatment irrespective of the dose applied had no stress-protective effect on the CSC-induced splenomegaly found in the VEH group. This lack of effect might be due to the fact that the increase in spleen weight during CSC exposure seems to be rather dependent on the number of bite wounds the CSC mice receive from the residents than due to the chronic psychosocial stressor exposure itself [29,33]. However, bite wounds were not assessed in our mice of the present study.

The analysis of HPA axis-related parameters further supported a stress-protective phenotype in mice chronically treated with XAP044. The CSC-induced increase in pituitary weight found in the VEH group was abolished in mice treated with XAP044, irrespective of the dose applied. Moreover, XAP044 at a dose of 100 µM was able to block the CSC-induced increase in adrenal weight, as well as the CSC-induced reduction in adrenal ACTH responsiveness *in vitro*. These parameters are reliable indicators for chronic psychosocial stress [30,34].

Together, these findings indicate that mice treated with XAP044, at least at a dose of 100 µM, are less vulnerable to physiological consequences as well as weight changes in lymphatic organs during chronic psychosocial stressor exposure.

### 4.2. Chronic Pharmacological mGlu7 Blockade Reverses CSC-Induced Innate but Not Physiological Anxiety

CSC exposure also reliably results in an increase in innate anxiety recorded, e.g., in the elevated platform, in the open-field, and in the light-dark box tests [20,21,31,35]. In addition, we were able to show previously that 19 days of CSC also induces an increase in physiological anxiety assessed in the SIH test [23]. These CSC effects were also found in CSC mice of the VEH group of the present study. They showed both a CSC-induced increase in innate anxiety, indicated by the reduced time spent in the BC of the LDB, as well as an increase in physiological anxiety, indicated by an increased stress-induced hyperthermic response in the SIH test. Interestingly, chronic treatment with XAP044 at a dose of 10 µM and 100 µM was able to block the CSC-induced increase in innate anxiety in the LDB. However, other than expected due to the stress-protective effects of XAP044 with respect to the physiological parameters discussed before, XAP044 treatment was not able to block the CSC-induced increase in physiological anxiety. All CSC groups showed an increase in the SIH response irrespective of treatment. It has to be mentioned that innate anxiety in the LDB was assessed on day 15, whereas the SIH test was performed on day 18 of CSC exposure. Therefore, the lack of effect of XAP044 treatment with respect to physiological anxiety might be explained by a sub-maximal/insufficient mGlu7 receptor saturation on day 18 of CSC, possibly due to a partial loss of pump functioning in the final stage of CSC exposure.

Altogether, the results of the present study suggest that mice chronically treated with XAP044 are dose-dependently protected against important CSC-induced alterations in the physiological, lymphatic, and anxiety parameters, at least with respect to innate anxiety. Therefore, it seems that the chronic pharmacological blockade of mGlu7, in addition to the genetic ablation, also results in a stress-resilient phenotype. Thus, it prevents a broad variety of maladaptive consequences of chronic psychosocial stressor exposure. However, when comparing the present results with our previous study in mGlu7-deficient mice [22], it seems that the chronic stress-protective phenotype is much more robust in mice with genetic ablation than in mice with pharmacological blockade of mGlu7 using XAP044. A less robust effect of XAP44 treatment compared to mGlu7 deficiency in mice was already obvious in the previously performed tests for acute stress and anxiety [15]. These differences in efficacy might be explained by the known drawbacks, with respect to the pharmacokinetic profile of the substance. In mice, XAP044 showed a high total blood clearance (76 mL/min/kg) and a very short apparent terminal half-life (0.4 h). Moreover, PK studies also revealed high binding to mouse and rat plasma protein, as well as rat brain homogenate, resulting in low concentrations of free XAP044 in brain and plasma [15]. Together, these characteristics of XAP044 may lead to a receptor saturation and, hence, mGlu7 blockade sufficient to induce a stress-protective phenotype, but with the effects being less pronounced than in mGlu7-defcient mice. However, these disadvantages can likely be overcome with future chemical derivatives of XAP044.

At this point, we can only speculate on how the inhibition of mGlu7 receptor activity by XAP044 can result in chronic stress-protective effects. At the single neuron level, it became clear that mGlu7 is expressed close to the presynaptic active zone and depending on the type of neuron, is capable of (down)-regulating L-glutamate, GABA and possibly also norepinephrine release [16,36]. Moreover, L-glutamate has a very low affinity at mGlu7, which possibly keeps the receptor inactive under many physiological situations [37]. The conditions of excessive neuronal glutamate release, possibly due to stressful events, may lead to the activation of mGlu7 and consequently to the inhibition of transmitter release [38]. Although XAP044 is expected to reduce receptor-mediated inhibitory control, the net outcome of this disinhibition will depend on the type of neuron and may differ between brain regions. In addition, the central pharmacological blockade of mGlu7 might induce changes in excitatory and inhibitory neurotransmitter release through altered negative feedback regulation, leading to a stress-resilient phenotype. However, the receptor’s widespread distribution in brain regions critical for emotion behaviors and for the regulation of stress responses [7,39], as well as potential downstream effects on other neurotransmitter systems, such as the dopamine and serotonin systems [40] that are known to alter e.g., anxiety-related behavior, makes it a challenge to understand whether the effects are region-specific and if they are based on changes in a specific transmitter system. One possibility is that the pharmacological blockade of mGlu7 may affect the dorsal raphe nucleus serotonin system and connected forebrain circuits, which are key central systems that are supposed to modulate anxiety- and stress-related behaviors and determine behavioral coping responses. This may result in a shift from reactive to proactive coping during CSC exposure [41,42,43]. Importantly, there is clear evidence from human and rodent studies that the way of behavioral coping predicts stress vulnerability and that there is a lowered risk to develop stress-related affective, as well as somatic diseases in proactively coping individuals [44,45]. However, with respect to pharmacological mGlu7 blockade and CSC exposure, this possibility is so far just speculative and has to be addressed experimentally in future studies.

## 5. Conclusions

Although the underlying mechanisms still have to be elucidated, the findings of the present study clearly show a chronic stress-protective effect in mice exposed to CSC upon treatment with the orthosteric-like mGlu7-selective antagonist XAP044. Thus, in addition to genetic mGlu7 ablation, pharmacological mGlu7 blockade is also able to induce a stress-resilient phenotype in mice, with respect to chronic psychosocial stress. This is an important finding, as it is much more relevant to human disorders and suggests pharmacological mGlu7 blockade as an innovative option to treat chronic stress-related diseases in clinical situations.

## Data Availability

Not applicable.

## Supplementary Materials

The following are available online at https://www.mdpi.com/article/10.3390/cells11111817/s1, Figure S1: Chronic administration of Ringer’s solution, vehicle and 100 µM of XAP044 in naïve mice.
