# Supplementary material for: Blocking Metabotropic Glutamate Receptor Subtype 7 via the Venus Flytrap Domain Promotes a Chronic Stress-Resilient Phenotype in Mice
Source: Cells. 2022 Jun 2;11(11):1817. doi: 10.3390/cells11111817 (PMC9180111; doi:10.3390/cells11111817)
Supplement: Supplementary file 1 [file cells-11-01817-s001.zip › cells-1716531-supplementary.pdf]

SUPPLEMENTAL FIGURE S1

A Experimental design

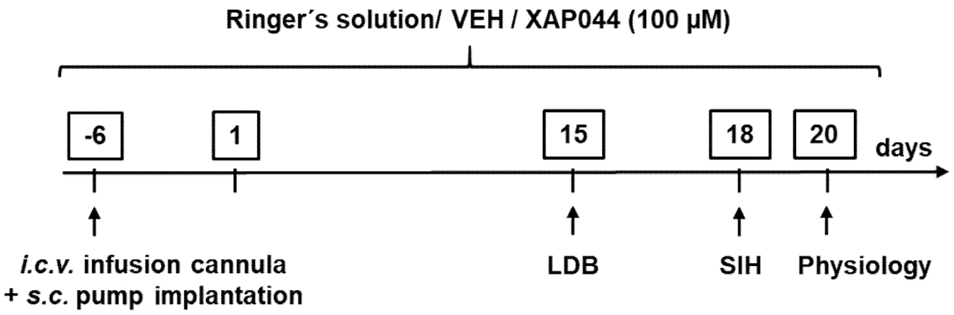

B

	Ringer´s solution	VEH (5% DMSO)	XAP044 (100 µM)
innate anxiety (LDB, % of time in BC), *n* = 9-11	51.77 ± 3.75	45.83 ± 4.53	44.99 ± 7.38
locomotion (LDB, number of line crossings/ time in BC), *n* = 9-11	0.57 ± 0.04	0.63 ± 0.04	0.55 ± 0.03
SIH (°C; T2-T1), *n* = 6-8	0.87 ± 0.22	0.80 ± 0.19	0.84 ± 0.16
body weight gain (g), *n* = 8-11	1.55 ± 0.40	2.11 ± 0.34	2.64 ± 0.16
thymus weight (mg), *n* = 8-11	48.84 ± 3.41	50.76 ± 1.38	52.79 ± 2.28
spleen weight (mg) *n* = 8-11	68.17 ± 2.33	72.22 ± 3.02	67.30 ± 1.02
pituitary weight (mg), *n* = 6-8	1.33 ± 0.05	1.40 ± 0.11	1.30 ± 0.04
adrenal weight (mg), *n* = 8-11	3.65 ± 0.03	3.61 ± 0.04	3.56 ± 0.08
plasma CORT (ng/ml), *n* = 8-11	35.65 ± 3.88	31.97 ± 1.54	28.38 ± 4.87

Figure S1. (**A**) Schematic illustration of the experimental design of chronic (*i.e.* 26 days) administration of Ringer´s solution, vehicle (VEH, 5% DMSO in Ringer´s solution), and XAP044 at a dose 100 µM via *i.c.v.* infusion cannulas connected to micro-osmotic pumps (*s.c.*) in naïve mice to test for possible undesirable effects on parameters typically assessed following CSC exposure. (**B**) Neither VEH nor XAP044 treatment had any adverse effects on the parameters assessed, when compared to mice treated with Ringer´s solution only. Data represent mean ± SEM. One-way ANOVA followed by Bonferroni *post hoc* testing. Abbreviations: LDB, light dark box; BC, bright compartment; SIH, stress-induced hyperthermia; CORT, corticosterone;
